# Supplementary material for: Long-term viable chimeric nephrons generated from progenitor cells are a reliable model in cisplatin-induced toxicity
Source: Commun Biol. 2023 Oct 28;6:1097. doi: 10.1038/s42003-023-05484-9 (PMC10613230; doi:10.1038/s42003-023-05484-9)
Supplement: Supplementary file 2 — Supplementary Information [file 42003_2023_5484_MOESM2_ESM.pdf]

Long-term viable chimeric nephrons generated from progenitor cells are a reliable model in cisplatin-induced toxicity

Kenji Matsui[1], Shuichiro Yamanaka[1,5]*, Sandy Chen[1], Naoto Matsumoto[1], Keita Morimoto[1], Yoshitaka Kinoshita[1,2], Yuka Inage[1,3], Yatsumu Saito[1], Tsuyoshi Takamura[1], Toshinari Fujimoto[1], Susumu Tajiri[1], Kei Matsumoto[1], Eiji Kobayashi[4], and Takashi Yokoo[1, 5]

[1]Division of Nephrology and Hypertension, Department of Internal Medicine, The Jikei University School of Medicine, Tokyo 105-8461, Japan
[2]Department of Urology, Graduate School of Medicine, The University of Tokyo, Tokyo 113-8654, Japan.
[3]Department of Pediatrics, The Jikei University School of Medicine, Tokyo 105-8461, Japan
[4]Department of Kidney Regenerative Medicine, The Jikei University School of Medicine, Tokyo 105-8461, Japan
[5]These authors jointly supervised this work

*Email: shu.yamanaka@jikei.ac.jp

Contents

Supplementary Figure 1: Immunostaining of the neonatal chimeric nephrons generated from EGFP mouse renal progenitor cells, associated with Figure 2
Supplementary Figure 2: Single-cell RNA-sequencing-related data, associated with Figure 3
Supplementary Figure 3: Limited maturation of *in vitro* renal organoids
Supplementary Figure 4: Rejection to EGFP antigen by hosts is weaker in neonates than in adults
Supplementary Figure 5: Rejection occurs in neonatal immunocompetent mice against rat renal progenitor cells, associated with Figure 7
Supplementary Figure 6: Modifications to help the survival and maturation of the human nephron progenitor cells injected in the neonatal kidney, associated with Figure 8

Supplementary Table 1: Comparative analysis of techniques for human nephron progenitor cell injection to neonatal mouse kidneys
Supplementary Table 2: List of primary antibodies for immunostaining
Supplementary Table 3: List of primers for reverse-transcription quantitative polymerase chain reaction (RT-qPCR)

Supplementary Figure 1

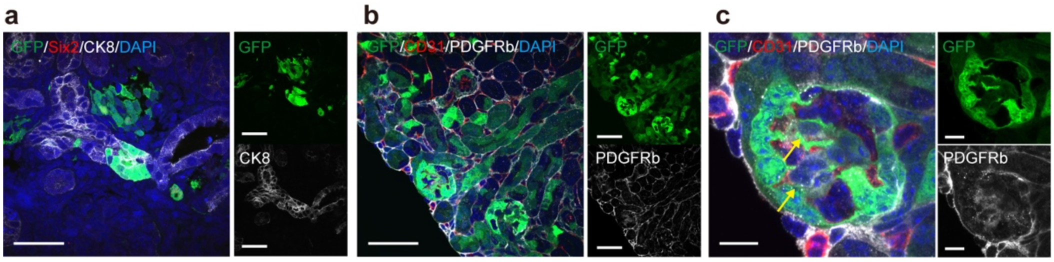

Supplementary Figure 1. Immunostaining of the neonatal chimeric nephrons generated from EGFP mouse renal progenitor cells, associated with Figure 2.
(**a**) Two days after the injection. Chimeric collecting ducts (CK8+, GFP+), which are derived from ureteric buds, are also observed in a limited area. (**b**) Two weeks after the injection, chimeric mesangial cells (yellow arrows; PDGFRb+, GFP+) derived from stromal progenitor cells are observed in the glomeruli. (**c**) Magnified images of (b). Scale bars, 50 μm in (a) and (b), 10 μm in (c). CK8, cytokeratin 8; DAPI, 4',6-diamidino-2-phenylindole; GFP, green fluorescent protein; Six2, sine oculis homeobox homolog 2

Supplementary Figure 2

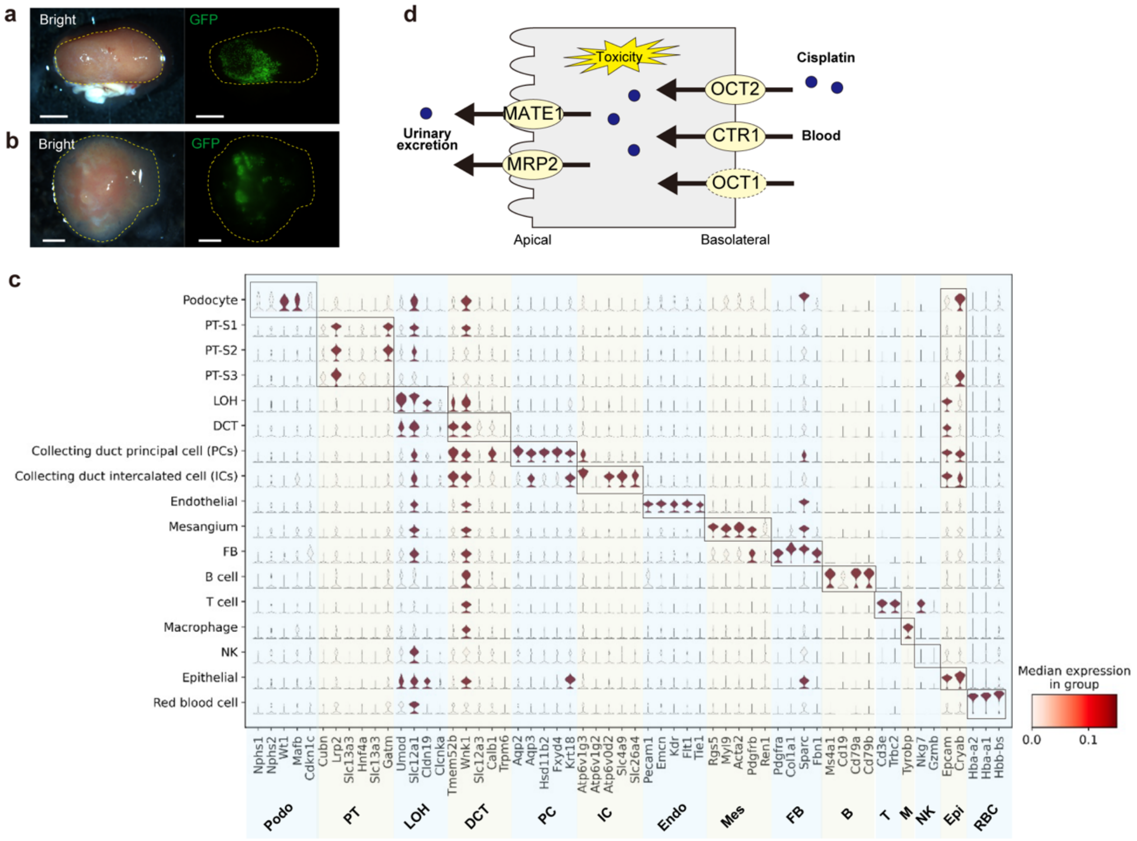

Supplementary Figure 2. Single-cell RNA-sequencing-related data, associated with Figure 3. (**a**) Host left kidney containing chimeric nephrons. (**b**) A cell mass with the perimeter trimmed around GFP (+) areas in the outer cortical layer of (a) and cutting with a scalpel. The boundaries are marked by yellow dotted lines in (a) and (b). (**c**) Violin plots showing expression levels of marker genes in each cluster. (**d**) A schematic of the transporters involved in cisplatin uptake and excretion in proximal tubular cells. Scale bars, 2 mm in (a) and 1 mm in (b). CIS, cisplatin; DCT, distal convoluted tubule; Endo, endothelial cell; Epi, epithelial cell; FB, fibroblast; GFP, green fluorescent protein; IC, collecting duct intercalated cell; LOH, loop of Henle; M, Macro, macrophage; Mes, mesangial cell; NK, natural killer cell; PC, collecting duct principal cell; Podo, podocyte; PT, proximal tubule; PT-S1~3, PT-segments 1–3; RBC, red blood cell.

Supplementary Figure 3

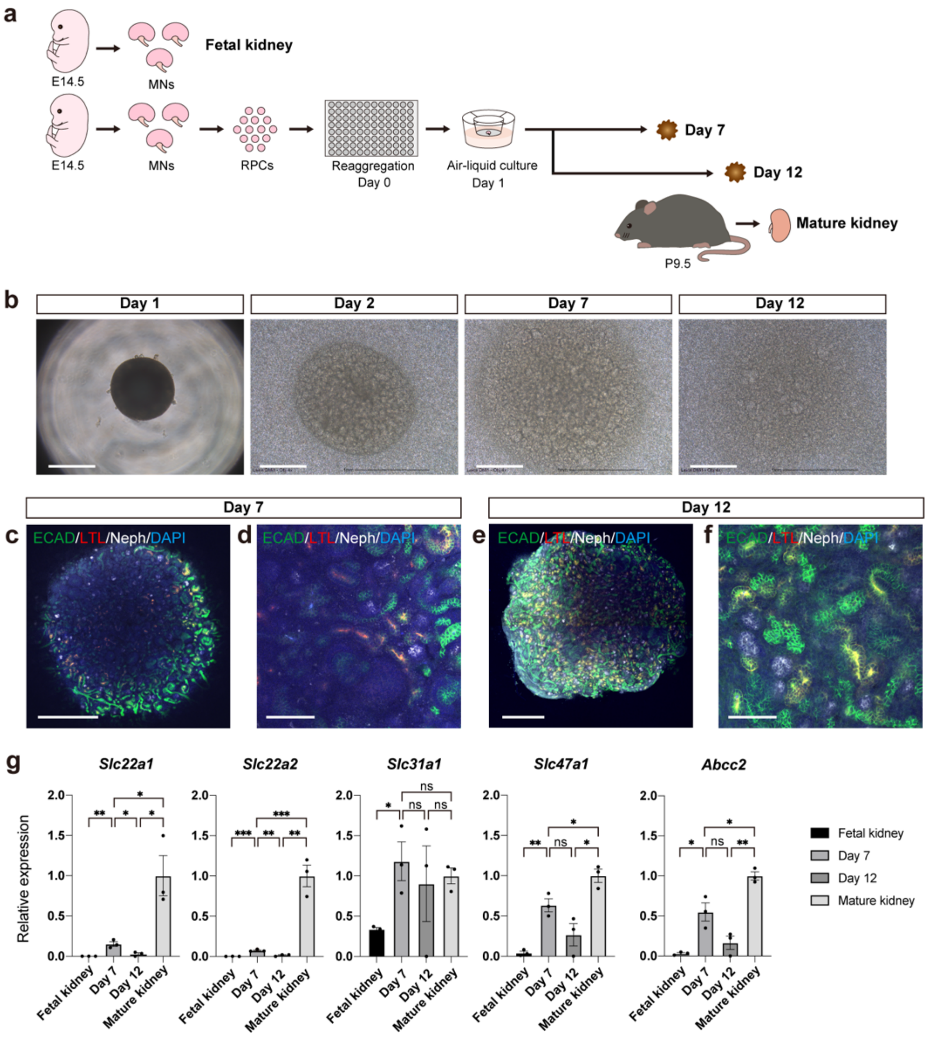

Supplementary Figure 3. Limited maturation of *in vitro* renal organoids. (**a**) A schematic of the experimental procedure. Fetal kidneys from E14.5 fetal mice, and mature kidneys from P9.5 mice that are equivalent in the developmental stage if E14.5 RPCs were cultured for 14 days, were used as controls. (**b**) Microscopic images of the renal spheroids over time. (**c-f**) Immunostaining images of the spheroids on days 7 (c, d) and 12 (e, f). Nephron structures including proximal tubules are formed. (**g**) The mRNA expression levels of proximal tubule transporters relative to glyceraldehyde 3-dehydrogenase among fetal kidneys, spheroids at days 7 and 12, and mature kidneys, in which the mean value for mature kidneys is set to 1.0 (n = 3 biologically independent samples). Error bars represent mean ± SEM. Data were analyzed using the two-tailed unpaired t -test. Scale bars, 500 μm in (b), (c), and (e) and 100 μm in (d) and (f). ECAD, E-cadherin; DAPI, 4’,6-diamidino-2-phenylindole; LTL, lotus tetragonolobus lectin; Neph, nephrin.

Supplementary Figure 4

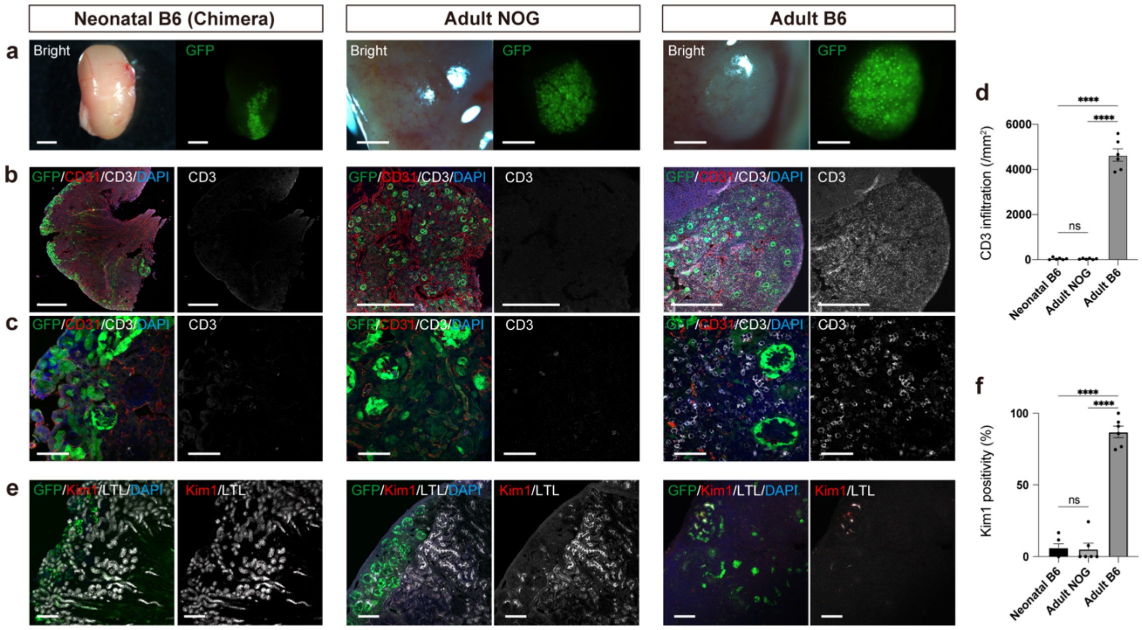

Supplementary Figure 4. Rejection to EGFP antigen by hosts is weaker in neonates than in adults. (**a**) Fluorescence stereomicroscopic images of chimeric nephrons regenerated from renal progenitor cells injected into neonates (left) and renal spheroids transplanted to adult NOG (middle) or B6 (right) mice collected after 2 weeks. The organoids transplanted to adult B6 mice are whitish and swollen compared with those transplanted to adult NOG mice. (**b, c**) Immunostaining images of (a) showing CD3-positive T lymphocyte infiltration. (**d**) Quantitative comparison of T lymphocyte infiltration between the groups (n = 6 sections from 3 biologically independent samples). Error bars represent mean ± SEM. Data were analyzed using the two-tailed unpaired t test. ns, not significant. **** $P < 0.0001$. (**e**) Immunostaining images of (a) showing damage to proximal tubule cells of exogenous nephrons by kidney injury molecule 1. (**f**) Quantitative comparison of the Kim1 positivity rate of exogenous proximal tubule cells between groups (n = 6 sections from 3 biologically independent samples). Error bars represent mean ± SEM. Data were analyzed using the two-tailed unpaired t test. ns, not significant. **** $P < 0.0001$. Scale bars, 1 mm in (a), 500 μm in (b), 50 μm in (c), and 100 μm in (e). DAPI, 4',6-diamidino-2-phenylindole; GFP, green fluorescent protein; LTL, lotus tetragonolobus lectin; Kim1, kidney injury molecule 1.

Supplementary Figure 5

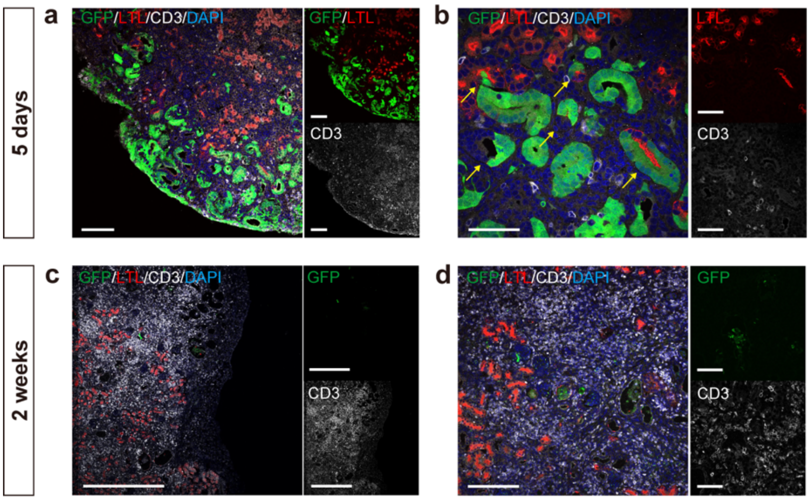

Supplementary Figure 5. Rejection occurs in neonatal immunocompetent mice against rat renal progenitor cells, associated with Figure 7. (**a, b**) Immunostaining of the chimeric nephrons regenerated from the renal progenitor cells (RPCs) of EGFP rats in neonatal B6 mice 5 days after injection. Chimeric tubular structures are intact while there are a few CD3-positive T lymphocytes. (**c, d**) Immunostaining of those 2 weeks after injection. No significant GFP-positive structures remain, and severe CD3-positive lymphocyte infiltration is apparent. Scale bars, 100 μm in (a), 50 μm in (b) and (d), and 500 μm in (c). DAPI, 4',6-diamidino-2-phenylindole; GFP, green fluorescent protein; LTL, lotus tetragonolobus lectin.

Supplementary Figure 6

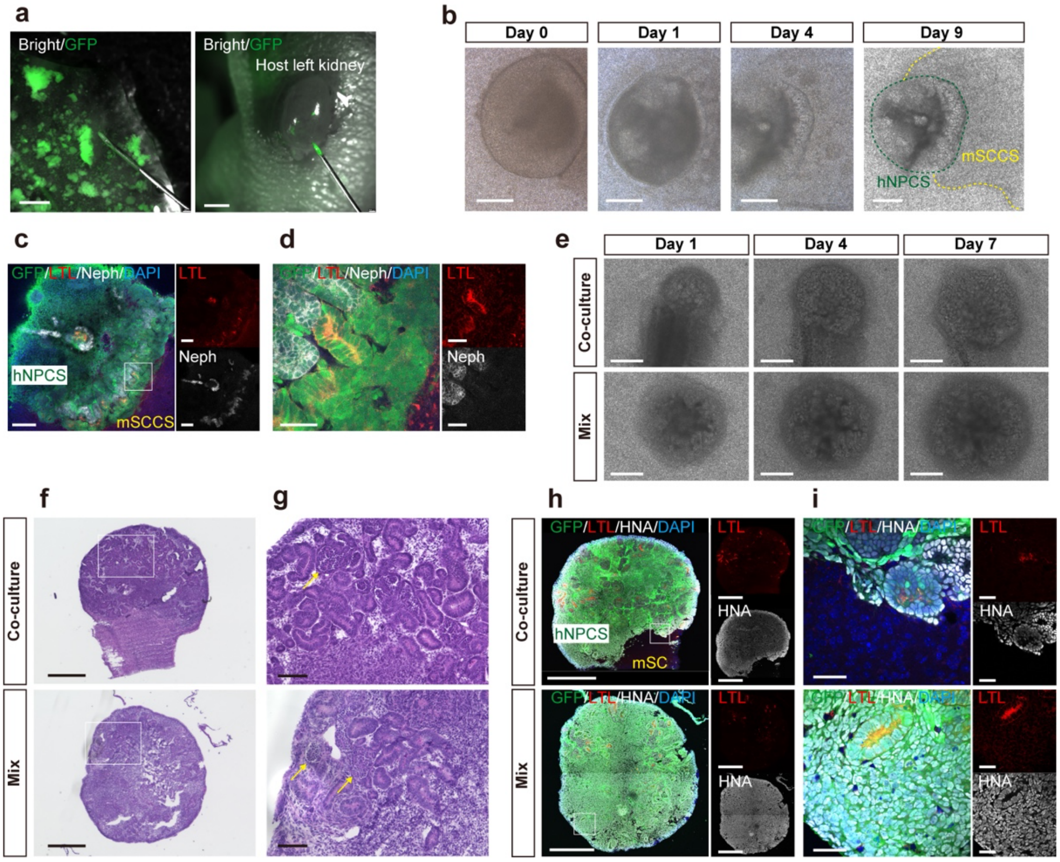

Supplementary Figure 6. Modifications to help the survival and maturation of the human nephron progenitor cells injected in the neonatal kidney, associated with Figure 8. (**a**) Human nephron progenitor cell spheres (hNPCS) broken up into clusters by manual pipetting (left) and those aspirated with 34G Hamilton syringe before injection (right). (**b**) hNPCS cultured in contact with the dissociated and re-aggregated mouse spinal cord cell sphere (mSCCS) for 9 days. (**c, d**) Immunostaining images of (b). The epithelialization of human NPCs is promoted especially in the proximity of the mSCCS. (**e**) hNPCS co-cultured with a piece of the fetal mouse spinal cord (Co-culture) and the mixed sphere of human NPCs and mouse spinal cord cells at a ratio of 1:1 (Mix) cultured for 7 days. (**f, g**) Hematoxylin–eosin staining images of (e). Nephrons consisting of glomeruli (yellow arrows) and tubules are observed in both groups. (**h, i**) Immunostaining images of (e). In the Co-culture sphere, mouse spinal cord (mSC, HNA-) cells are in contact with the human nephrons (HNA+). In the Mix sphere, mSC cells are scattered, but human nephron formation is not inhibited. Scale bars, 2 mm in (a), 500 µm in (b) and (e), (f), and (h); 200 µm in (c); 50 µm in (d) and (i); and 100 µm in (g). GFP, green fluorescent protein; DAPI, 4',6-diamidino-2-phenylindole; HNA, human nuclear antigen; LTL, lotus tetragonolobus lectin; Neph, nephrin.

Supplementary Table 1: Comparative analysis of techniques for human nephron progenitor cell injection to neonatal mouse kidneys

	Dissociated cells without spinal cord cells	Dissociated cells with spinal cord cells	Clusters without spinal cord cells	Clusters with spinal cord cells
Percentage of experiments with confirmed donor proximal tubule formation	0% (0 of 4)	0% (0 of 3)	0% (0 of 10)	32% (6 of 19)

Supplementary Table 2. List of primary antibodies for immunostaining

Antibodies	Source	Identifier	Host	Dilution
CK8(TROMA- I)	DSHB	N/A	Rat	1:100
Six2	Proteintech	Cat. # 11562-1-AP	Rabbit	1:100
GFP	Abcam	Cat. # ab13970	Chicken	1:200
Nephrin	Progen	Cat. # GP-N2	Guinea pig	1:100
CD31	R&D systems	Cat. # AF3628	Gout	1:100
LTL	Vector	Cat. # B-1325	Biotinylated	1:200
ECAD	Cell Signaling Technology	Cat. # 3195S	Rabbit	1:100
CTR1	Abcam	Cat. # ab129067	Rabbit	1:100
MATE1	Cell Signaling Technology	Cat. # 14550S	Rabbit	1:100
Mouse Kim1	R&D systems	Cat. # AF1817	Gout	1:100
Rat Kim1	R&D systems	Cat. # AF3689	Gout	1:100
Human Kim1	R&D systems	Cat. # AF1750	Gout	1:100
CD3	Abcam	Cat. # ab5690	Rabbit	1:100
HNA	Millipore	Cat. # MAB1281	Mouse	1:100
PDGFRb	R&D systems	Cat. # AF1042	Gout	1:100

CK8, cytokeratin 8; ECAD, E-cadherin; GFP, green fluorescent protein; HNA, human nuclear antigen; Kim1, kidney injury molecule 1; LTL, lotus tetragonolobus lectin; Six2, sine oculis homeobox homolog 2.

Supplementary Table 3. List of primers for reverse-transcription quantitative polymerase chain reaction (RT-qPCR)

Reagent	Source	Identifier
TaqMan Gene Expression Assays (*GAPDH*)	Thermo Fisher Scientific	Cat. # Mm99999915_g1
TaqMan Gene Expression Assays (*Slc22a1*)	Thermo Fisher Scientific	Cat. # Mm00456303_m1
TaqMan Gene Expression Assays (*Slc22a2*)	Thermo Fisher Scientific	Cat. # Mm01313508_m1
TaqMan Gene Expression Assays (*Slc31a1*)	Thermo Fisher Scientific	Cat. # Mm00558247_m1
TaqMan Gene Expression Assays (*Slc47a1*)	Thermo Fisher Scientific	Cat. # Mm00840361_m1
TaqMan Gene Expression Assays (*Abcc2*)	Thermo Fisher Scientific	Cat. # Mm00496899_m1

GAPDH, glyceraldehyde 3-dehydrogenase.
