# Supplementary material for: Long-term viable chimeric nephrons generated from progenitor cells are a reliable model in cisplatin-induced toxicity
Source: Commun Biol. 2023 Oct 28;6:1097. doi: 10.1038/s42003-023-05484-9 (PMC10613230; doi:10.1038/s42003-023-05484-9)
Supplement: Supplementary file 3 — Description of Supplementary Materials [file 42003_2023_5484_MOESM3_ESM.docx]

**Description of Additional Supplementary Files**

**File name:** Supplementary Data 1

**Description:** The source data behind the graphs in the paper

**File name:** Supplementary Movie 1

**Description:** Surgical video of the neonatal niche injection method
